# Sponsorship bias and quality of randomised controlled trials in veterinary medicine

**DOI:** 10.1186/s12917-017-1146-9

**Published:** 2017-08-14

**Authors:** K. J. Wareham, R. M. Hyde, D. Grindlay, M. L. Brennan, R. S. Dean

**Affiliations:** 1 Centre for Evidence-based Veterinary Medicine, School of Veterinary Medicine and Science, The University of Nottingham, Sutton Bonington campus, Loughborough, LE12 5RD UK; 2 Centre of Evidence-based Dermatology, University ofNottingham, Kings Meadow campus, Lenton Lane, Nottingham, NG7 2NR UK

**Keywords:** Clinical trials, Study design and data analysis, Evidence based medicine, Risk of bias

## Abstract

**Background:**

Randomised controlled trials (RCTs) are considered the gold standard form of evidence for assessing treatment efficacy, but many factors can influence their reliability including methodological quality, reporting quality and funding source.

The aim of this study was to examine the relationship between funding source and positive outcome reporting in veterinary RCTs published in 2011 and to assess the risk of bias in the RCTs identified.

**Methods:**

A structured search of PubMed was used to identify feline, canine, equine, bovine and ovine clinical trials examining the efficacy of pharmaceutical interventions published in 2011. Funding source and outcomes were extracted from each RCT and an assessment of risk of bias made using the Cochrane risk of bias tool.

**Results:**

Literature searches returned 972 papers, with 86 papers (comprising 126 individual RCTs) included in the analysis. There was found to be a significantly higher proportion of positive outcomes reported in the pharmaceutical funding group (P) compared to the non-pharmaceutical (NP) and ‘no funding source stated’ (NF) groups (*P* = 56.9%, NP = 34.9%, NF = 29.1%, *p* < 0.05). A high proportion of trials had an unclear risk of bias across the five criteria examined.

**Conclusions:**

We found evidence that veterinary RCTs were more likely to report positive outcomes if they have pharmaceutical industry funding or involvement. Consistently poor reporting of trials, including non-identification of funding source, was found which hinders the use of the available evidence.

**Electronic supplementary material:**

The online version of this article (doi:10.1186/s12917-017-1146-9) contains supplementary material, which is available to authorized users.

## Background

In order to effectively practice veterinary medicine in an evidence-based way, it is imperative that accurate scientific evidence is available so that the evidence base is complete, reliable, and therefore not misleading. Randomised controlled trials (RCTs), along with their synthesis in the form of systematic reviews, are considered to be the gold standard method for assessing the efficacy of treatment interventions and are a valuable source of information on which to base clinical decisions [1]. The results of RCTs can however be affected by many biases including selection, performance, detection, attrition and reporting biases [2, 3]. The presence of bias can lead to misinterpretation of treatment efficacy or harms, and mislead clinicians when putting the evidence into practice.

Sponsorship bias (the influence of funding source on the reporting of trial results) is an additional potential problem when assessing the reliability of RCTs. The medical literature contains differing reports over whether financial conflicts of interest influence the reported results of a trial. Some studies report a greater likelihood of positive results for industry funded trials [4, 5], while some report no difference between industry and non-industry sponsored trials [6, 7]. A recent overview of medical literature in a Cochrane systematic review concluded that drug and medical device studies were more likely to report favourable results when the study was sponsored by a manufacturer [8].

There have been several studies examining the methodological and reporting quality of clinical trials in the published veterinary literature [9–11]. Such studies have highlighted issues with the reporting of RCTs and have shown how these reporting deficiencies are associated with an increased likelihood of a trial reporting one or more positive outcomes [10]. To our knowledge, no studies to date have examined the influence of funding source on the likelihood of reporting positive outcomes in the veterinary RCT literature.

The aim of this study was to examine the relationship between funding source and proportions of positive outcome reporting in veterinary RCTs involving a pharmaceutical intervention published in a single calendar year (2011). A secondary aim was to assess the risk of bias of veterinary RCTs published in the same time period.

## Methods

A cross-sectional study of veterinary RCTs was conducted. The target population was feline, canine, equine, bovine and ovine RCTs where a pharmaceutical agent was the intervention of interest and efficacy was assessed. The sample population was feline, canine, equine, bovine and ovine RCTs published in 2011 within journals indexed in PubMed.

### Search strategy and filtering of results

**Fig. 1:**
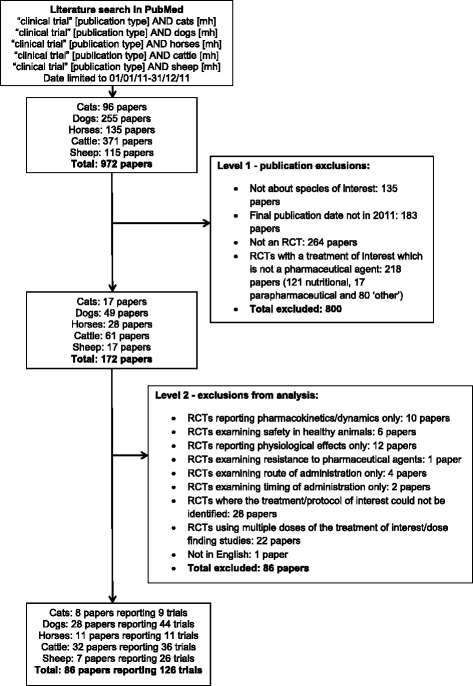
Summary of the number of papers retrieved from literature searches, numbers of papers excluded using Level 1 and 2 exclusion criteria and number of papers and individual trials analysed for each species and overall

A structured search of PubMed was conducted in June 2013 using the “clinical trial” Publication Type combined with the relevant species MeSH heading e.g. “clinical trial” [publication type] AND cats [mh]. This was done for each of the 5 species studied: cats, dogs, horses, cattle and sheep (Fig. 1). The search was limited to one calendar year with a PubMed filter: 01/01/11–31/12/11. Search results were exported into EndNote® software for filtering. Papers indexed as RCTs by PubMed (“randomised controlled trials” [publication type]) were extracted, investigators then confirmed if they were RCTs according to the Cochrane definition below (http://www.cochrane.org/glossary/):

**“**An experiment in which two or more interventions, possibly including a control intervention or no intervention, are compared by being randomly allocated to participants. In most trials one intervention is assigned to each individual but sometimes assignment is to defined groups of individuals (for example, in a household) or interventions are assigned within individuals (for example, in different orders or to different parts of the body).”All publications containing trials confirmed by the investigators as being RCTs, published in 2011, and relevant to the species of interest were then categorised into four intervention subcategories based on the main intervention of interest of the study (Table 1 - Level 1 exclusion criteria):

1. Pharmaceutical – consisting of an active pharmaceutical ingredient, including anthelmintics and vaccines
2. Nutritional
3. Para-pharmaceutical – including probiotics, prebiotics, synbiotics, nutraceuticals and supplements/vitamins/minerals if not considered part of the total dietary ration
4. Other – including surgical interventions, management/husbandry interventions, non-medicinal shampoos, studies relating to diagnostic tests.

**Table 1:** Two levels of inclusion and exclusion criteria applied to the search results

Level 1: Inclusion criteria for publications	Level 1: Exclusion criteria for publications
Species of interest is cats, dogs, horses, cattle or sheep	Not about cats, dogs, horses, cattle or sheep
Published in 2011	E published only in 2011 if full publication occurred in a different calendar year
RCT according to PubMed publication types and the Cochrane definition	Not an RCT (not indexed as an RCT by PubMed or not fulfilling Cochrane definition of an RCT)
Treatment of interest is a pharmaceutical intervention (including anthelmintics and vaccines)	Treatment of interest is not a pharmaceutical agent e.g. nutritional, surgical, animal husbandry etc
Level 2: Inclusion criteria for analysis of pharmaceutical RCTs	Level 2: Exclusion criteria for analysis of pharmaceutical RCTs
Primary aim is to assess efficacy	Primary aim was not to assess efficacy (pharmacokinetic/dynamic studies, safety studies, physiological effects, resistance testing, testing routes of administration only, testing timing of administration only)
Identifiable treatment or protocol of interest	Treatment or protocol of interest could not be identified
Single dose of the treatment of interest used	Multiple doses of the treatment of interest used/dose finding studies
Published in English	Not available in English

Only publications within the ‘Pharmaceutical intervention’ subcategory were included in this study; these were assessed for further eligibility for analysis according to the second level of inclusion and exclusion criteria in Table 1.

Publications included in the analysis were therefore single dose efficacy studies of pharmaceutical interventions in cats, dogs, horses, cattle or sheep published in 2011. In the case of a publication containing more than one trial, each trial was included independently in the analysis if it met all inclusion criteria.

### Sources of funding

For each included trial the source of funding was categorised as one of the following:

1. Pharmaceutical company funding stated or pharmaceutical company involvement (e.g. drug donated by a pharmaceutical company or authors associated with a pharmaceutical company) (P)
2. Non-pharmaceutical company funding stated (NP)
3. No funding source stated (NF)

### Outcome recording

All outcomes mentioned in the methods section of the manuscripts were extracted and the result for each outcome was recorded. Outcomes that were reported as results but not mentioned in the methods were not included in the analysis. The result for each outcome was recorded in one of the five categories below (adapted from [10]):

1. Treatment of interest had a statistically significant positive effect on the outcome

-  ▪ Treatment better than any control group
-  ▪ Treatment equal to positive control group (whether non-inferiority/equivalence design or not)
-  ▪ Safety/lack of adverse effects equal to, or better than, any control group

2. Treatment of interest had a statistically significant negative effect on the outcome

-  ▪ Treatment worse than any control group
-  ▪ Treatment equal to negative control group
-  ▪ Safety/adverse effects worse than any control group
-  ▪ Treatment equal to a positive control group in a superiority analysis

3. No significant difference between treatment and control groups

-  ▪ Outcome remained constant throughout the study (no measurable effect of treatment on the outcome)

4. Results for the outcome were described only

-  ▪ There was data reported for an outcome that could have been statistically analysed, but no analysis was presented (if an outcome did not occur in any group, e.g. adverse events, it was treated as having been statistically analysed)
-  ▪ Outcomes such as descriptions of pathological appearances with no numerical data attached.

5. Results for the outcome were not reported

Outcome measures that had multiple components (e.g. complete blood count and serum biochemistry, meat yield and meat quality grade assessments) were classed as a single outcome each unless specific features were relevant to the disease, in which case these were extracted as individual outcomes. If an outcome had a result recorded at multiple time points, an overall judgement was made as to which of the above categories was most appropriate (i.e. the outcome was only recorded once regardless of how many time points it was measured). Where multiple treatment and control groups were used, each group containing the treatment of interest (either alone or in combination) was compared to its relevant control group for each outcome.

### Risk of bias assessment

All the included studies were assessed at the study level using the Cochrane risk of bias tool [2]. The five features assessed were: random sequence generation, allocation concealment, blinding, incomplete outcome data and selective outcome reporting. Following the Cochrane guidelines for the risk of bias tool each category was assessed as being at a high, low or unclear risk of bias. These features allow the risks of selection bias, performance bias, detection bias, attrition bias and reporting bias to be assessed (see Additional file 1 for definitions of these types of bias). We did not include the category of ‘Other bias’ from the tool.

All assessments made throughout the study were agreed upon by two authors (KW and RH/RD) with any disputes resolved by a third author (RD/RH).

### Statistical analysis

Categorical data were presented descriptively as raw numbers and percentages. Associations between funding source and positive outcome reporting were analysed using a Pearson’s chi squared test and Bonferroni post hoc test with adjusted *p* values. Significance level was set at *p* < 0.05. Results for different species are described only and were not compared statistically due to small group sizes. All statistical analyses were conducted in IBM SPSS Version 21.

## Results

### Overall study numbers

A total of 972 papers were retrieved from the initial searches (96 for cats, 255 for dogs, 135 for horses, 371 for cattle and 115 for sheep; Fig. 1). Following an initial review and exclusions based on year of publication in paper copy and species of interest there were 410 papers given the Publication Type for RCTs in PubMed; 390 of which were confirmed to be RCTs according to the Cochrane definition. Of these, 172 papers (172/390, 44.1%) were describing RCTs in which the treatment of interest was a pharmaceutical intervention and were included in further analysis (Fig. 1). The remainder comprised nutritional studies (121/390, 31.0%), para-pharmaceutical agent studies (17/390, 4.4%) and ‘other RCTs’ (80/390, 20.5%).

Following application of the second set of exclusion criteria to the RCT pharmaceutical intervention studies, 86 papers remained in the study from which outcomes, bias and sources of funding were extracted (Fig. 1, Table 2 and Additional file 2: Table S1). Eleven papers (all except one of which were within the pharmaceutical funding group) reported more than one RCT, notably one sheep paper reported 19 separate RCTs. As each trial was assessed individually as a separate entry, there were 126 trials included in the full analysis (Table 2 and Additional file 3 for full references of the publications analysed).

**Table 2:** Number and funding source of papers and individual trials following level 2 exclusion criteria application

	Number of cat papers (trials)	Number of dog papers (trials)	Number of horse papers (trials)	Number of cattle papers (trials)	Number of sheep papers (trials)	Total number of papers (trials, % of total trials)
Papers including pharmaceutical agent RCTs	17	49	28	61	17	**172**
Papers excluded from analysis^a^	9	21	17	29	10	**86**
Papers analysed	8 (9 trials)	28 (44 trials)	11 (11 trials)	32 (36 trials)	7 (26 trials)	**86 (126 trials)**
Funding sources of analysed papers						
Pharmaceutical company funded/pharmaceutical company involvement	4 (5 trials)	17 (33 trials)	4 (4 trials)	20 (23 trials)	2 (21 trials)	**47 (86 trials; 68.3%)**
Non pharmaceutical funding stated	3 (3 trials)	4 (4 trials)	2 (2 trials)	6 (7 trials)	3 (3 trials)	**18 (19 trials; 15.1%)**
No funding stated	1 (1 trial)	7 (7 trials)	5 (5 trials)	6 (6 trials)	2 (2 trials)	**21 (21 trials; 16.7%)**

Included studies are the pharmaceutical agent RCTs. ^a^See Additional Table 1 for reasons for exclusions from analysis. There was no statistical difference (*p* = 0.53) between funding sources between companion animal species (cats, dogs and horses) and farm animal species (cows and sheep)

Of these 126 trials, 86 (68.3%) were funded by the pharmaceutical industry or had pharmaceutical company involvement, 19 trials (15.1%) explicitly stated they were not funded by the pharmaceutical industry, and 21 trials (16.7%) did not state any source of funding within the manuscript (Table 2).

### Funding source and outcome reporting

From the 126 trials included in the analysis, a total of 960 outcomes were extracted. Overall, 47.5% of outcomes (456/960) recorded in the trials were statistically positive compared to 28.8% (276/960) which were recorded as being statistically negative; 1.9% of outcomes (18/960) remained unchanged during the study (no significant difference category), 14.7% of outcomes (141/960) were described only and 7.2% (69/960) were not reported at all in the results (Table 3).

**Table 3:** Categorisation of individual outcomes from 126 trials (960 outcomes)

	Outcomes from trials with pharmaceutical funding/involvement	Outcomes from trials with non-pharmaceutical funding stated	Outcomes from trials with no funding source stated	Outcomes from all trials
Positive outcomes	56.9% (339/596)_a_	34.9% (66/189)_b_	29.1% (51/175)_b_	**47.5% (456/960)**
Negative outcomes	23.5% (140/596)_a_	37.6% (71/189)_b_	37.1% (65/175)_b_	**28.8% (276/960)**
No difference	0.8% (5/596)_a_	2.6% (5/189)_a,b_	4.6% (8/175)_b_	**1.9% (18/960)**
Described only	12.8% (76/596)	16.9% (32/189)	18.9% (33/175)	**14.7% (141/960)**
Not reported	6.0% (36/596)s	7.9% (15/189)	10.3% (18/175)	**7.2% (69/960)**

Data shown as percentages and raw numbers in brackets. Significant differences (*p* < 0.05) existing between funding categories within rows are indicated by differing subscript letters. (No subscript letters in a row signifiy no significant differences. The presence of a subscript letter (e.g. ‘a’ in a cell indicates that it is significantly different from a cell marked with a different letter (e.g. ‘b’). If a cell has two subscript letters (e.g. ‘a,b’) then it is different from cells individually marked with each letter)

Between funding groups there were significant differences in the proportions of outcomes recorded in each of the outcome categories (Table 3, Pearsons chi squared, *p* < 0.001). The proportion of positive outcomes reported was significantly higher in the pharmaceutical group than in the non-pharmaceutical and ‘no funding source stated’ groups (*P* = 56.9%, NP = 34.9%, NF = 29.1%, *p* < 0.05). Correspondingly, there was a significantly lower proportion of negative outcomes recorded for the pharmaceutical group compared to the other two groups (*P* = 23.5%, NP = 37.6%, NF = 37.1%, *p* < 0.05). Across all funding groups the proportion of outcomes recorded as ‘no significant difference’ was low, however the ‘no funding group’ had a significantly higher proportion compared to the pharmaceutical group (NF = 4.6%, *P* = 0.8%, *p* < 0.05); the non-pharmaceutical group was not different to either of the other two groups (NP = 2.6%, *p* > 0.05). There were no significant differences between the funding groups in the proportion of ‘described only’ or ‘not reported’ outcomes (*p* > 0.05).

The above analysis categorised a treatment group which had equal results to a positive control group as a ‘positive’ outcome, even if the study did not use a non-inferiority design. If these results were instead considered to be in a ‘no significant difference’ category, the pattern of significantly higher positive, and lower negative, outcome reporting in the pharmaceutical group compared to the other two groups was still present (*p* < 0.05).

### Risk of bias assessment

**Fig. 2:**
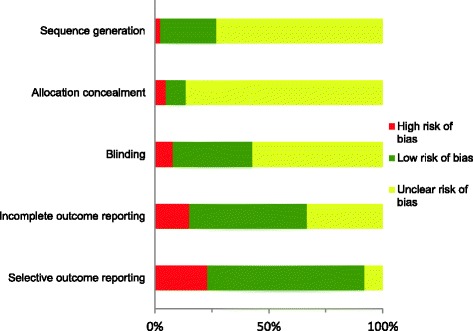
Percentages of all trials (*N* = 126) at high, low or unclear risk of bias for the five criteria assessed

Of the 126 included trials, the majority (92/126, 73.0%) were assessed as having an unclear risk of selection bias as there was inadequate or no description of how randomisation sequences were generated and employed. The vast majority of the trials were assessed as having an unclear risk of bias for allocation concealment (109/126, 86.5%) as it was impossible to determine what procedures had been followed. Blinding was reported more consistently, with 44 of the 126 trials (34.9%) being assessed as having a low risk of bias, 72/126 (57.1%) having an unclear risk, and the remaining 10 (7.9%) having a high risk of bias. Around half of the trials (65/126, 51.6%) were at low risk of bias for incomplete outcome reporting. There was a high risk of bias for incomplete outcome reporting in 19 out of the 126 trials (15.1%) due to missing data, or lack of analysis of the full population of animals randomised in the trial. Twenty-nine of the 126 trials (23.0%) were judged to be at a high risk of bias for selective outcome reporting, only 10/126 (7.9%) were at an unclear risk of bias, and the remaining 87 (69.0%) were assessed as being at a low risk of bias (Fig. 2 and Table 4).

**Table 4:** Risk of bias for trials within different funding categories and overall

	Risk of bias	Pharmaceutical funding/involvement (86 trials)	Non pharmaceutical funding declared (19 trials)	No funding source declared (21 trials)	All trials (126 trials)
Sequence generation	High	3 (3.5%)	0 (0%)	0 (0%)	**3 (2.4%)**
	Low	16 (18.6%)	7 (36.8%)	8 (38.1%)	**31 (24.6%)**
	Unclear	67 (77.9%)	12 (63.2%)	13 (61.9%)	**92 (73.0%)**
Allocation concealment	High	5 (5.8%)	1 (5.3%)	0 (0%)	**6 (4.8%)**
	Low	5 (5.8%)	3 (15.8%)	3 (14.3%)	**11 (8.7%)**
	Unclear	76 (88.4%)	15 (78.9%)	18 (85.7%)	**109 (86.5%)**
Blinding	High	5 (5.8%)	3 (15.8%)	2 (9.5%)	**10 (7.9%)**
	Low	29 (33.7%)	7 (36.8%)	8 (38.1%)	**44 (34.9%)**
	Unclear	52 (60.5%)	9 (47.4%)	11 (52.4%)	**72 (57.1%)**
Incomplete outcome reporting	High	14 (16.3%)	1 (5.3%)	4 (19.0%)	**19 (15.1%)**
	Low	36 (41.9%)	15 (78.9%)	14 (66.7%)	**65 (51.6%)**
	Unclear	36 (41.9%)	3 (15.8%)	3 (14.3%)	**42 (33.3%)**
Selective outcome reporting	High	18 (20.9%)	5 (26.3%)	6 (28.6%)	**29 (23.0%)**
	Low	64 (74.4%)	11 (57.9%)	12 (57.1%)	**87 (69.0%)**
	Unclear	4 (4.7%)	3 (15.8%)	3 (14.3%)	**10 (7.9%)**

Data expressed as as raw numbers and percentages of total trials

The results of comparing the quality criteria across the trials in different funding are shown in Table 4. The highest percentage of unclear risk for sequence generation was in the pharmaceutical group where 67 out of 86 trials (77.9%) were judged to be at an unclear risk of bias with a lower proportion in the non-pharmaceutical group (12/19, 63.2%) and 3/21 (61.9%) in the no funding declared group (13/21, 61.9%). The pharmaceutical group also had a higher proportion of unclear risk for incomplete outcome reporting in comparison to the other two funding groups (*P* = 36/86, 41.9%, NP = 3/19, 15.8%, NF = 3/21, 14.3%) and a correspondingly lower proportion of trials in the low risk category for this criteria. The high risk for selective outcome reporting was seen across all the funding categories (*P* = 18/86, 20.9%; NP = 5/19, 26.3%; NF = 6/21, 28.6%), however the pharmaceutical group had the largest proportion of studies in the low risk category for this criteria compared to the other groups (*P* = 64/86, 74.4%, NP = 11/19, 57.9%, NF = 12/21, 57.1%). Similar distributions of risk for blinding and allocation concealment were seen across the funding groups (Table 4).

## Discussion

This study found a significantly higher proportion of positive outcomes reported in RCTs with pharmaceutical funding (56.9%) or involvement compared to those with declared non-pharmaceutical funding (34.9%) or with no funding source stated (29.1%) within the sample of literature studied. There was a correspondingly lower proportion of negative outcomes reported in trials within the pharmaceutical funding group (23.5%) compared to the other two groups (37.6% and 37.1%). When assessing the trials for risk of bias across the five main categories using the Cochrane risk of bias tool, a large proportion were at an ‘unclear’ risk indicating significant reporting deficiencies. A high risk of bias was most predominantly seen for selective outcome reporting (reporting bias), and more moderately for incomplete outcome data (attrition bias) and blinding (detection bias). Proportions of trials at high, low or unclear risk of bias for the different quality criteria were largely similar across funding categories.

The sponsorship bias detected in this study is in accordance with many reports in the medical literature where an association between funding source and positive results has been demonstrated, most notably in a Cochrane Review of drug and medical devices [8]. There are many reasons why such a bias may be present in the published literature including differences in the methodological quality of trials; inherent biases in trial conduct to favour a treatment; a genuinely greater likelihood that pharmaceutical companies would be testing pharmaceutical agents that are likely to perform well; and inadequacies in trial reporting which favour a treatment. Additionally, publication bias may play a role through researchers within different environments potentially being more or less likely to publish trials demonstrating a positive effect compared to trials showing a ‘negative’ result. Further studies are required to examine this finding and its potential causative factors in more detail, in particular whether there are correlations between quality criteria and funding source, something which this study did not investigate.

There are a variety of methods that could have been utilised for the current study. For example, in medical literature reviewing the presence of sponsorship bias, it is common to report one overall conclusion for a paper (i.e. overall the paper has a positive/negative/not significantly different outcome) determined either by the reviewers, based on the assertions of the authors or on the statistical analysis of one primary outcome of the study [4, 7, 12]. The method we have used, whereby we have extracted each outcome and its result, is more achievable in the veterinary literature, as primary outcomes are often unspecified [10, 13], but different results would potentially be obtained using a different approach. Of note in this study is the potential for differences between species, and potential clustering of some types of trials, e.g. anthelmintic efficacy trials, to have skewed the data; these limitations will be discussed in more detail below. To date, we have found no other publications examining the association of funding source with positive outcome reporting in the veterinary literature with which to compare our results. The group of trials with no funding source stated are particularly difficult to assess in this study as no assumptions can be made as to which of the two other groups they would most appropriately belong to. Within the results, they appear to be most like the non-pharmaceutical group of trials in their characteristics, but this in itself highlights a continuing problem of poor reporting of clinical trials (20% of trials in this study did not report a funding source).

Selective outcome reporting, for example not reporting, or incompletely reporting, results for pre-specified outcomes, or reporting outcomes that were not pre-specified, can introduce reporting bias into a study and influence the overall results [2, 3]. A striking feature of our data was the high proportion of outcomes that were described only (18.9%) or were mentioned in the materials and methods then not reported in the results (10.3%). This could partly be due to manuscripts not detailing clearly which of the parameters being measured were intended to be outcomes used to assess efficacy, leading us to misclassify the information, highlighting again the issue of poor reporting. A previous study reporting quality criteria and outcome data from a sample of dog and cat trials also reported a high percentage of outcomes with no formal statistical analysis (31%) and a lower percentage not reported at all (3.1%) [10]. The proportions of outcomes in these two categories contribute to the overall high risk of reporting bias (selective outcome reporting) found in this study. Research has shown that outcomes that are not reported, or incompletely reported are more likely to be statistically insignificant [14, 15]. This highlights the need for pre-specified primary and secondary outcomes to be explicitly stated in the methods and adhered to when reporting results. One approach which should help to combat this problem is for all clinical trial protocols to be registered in advance, so a comparison can be made with the final report; this approach is being championed by the AllTrials campaign in human medicine. AllTrials aims to ensure that all clinical trials are registered before they commence and that all are fully reported [16] (www.alltrials.net). A similar initiative is currently underway for veterinary clinical trials [17]; these schemes should also help to combat publication bias. Publication bias, meaning negative studies are less likely to be published than positive ones, is a problem that has been identified across scientific publishing generally and which can lead to over estimates of treatment effects [3, 15]. The potential impact of publication bias on our study results would depend on who was funding any unpublished trials.

The high proportions of ‘unclear’ risk of bias for the five quality criteria assessed in this study indicate a significant issue with poor reporting, a feature which has also been described in previous quality assessments of veterinary clinical trial literature [9, 10, 13]. This does not necessarily equate to poor methodological trial conduct, but a lack of complete reporting means that the methodology cannot be adequately assessed [18, 19]. This study did not set out to assess the impact of risk of bias on levels of positive outcome reporting. However, it has previously been shown in both veterinary and medical literature that incomplete or inadequate reporting of certain quality criteria (e.g. method of randomisation) is linked to an exaggeration of treatment efficacy [10, 13, 20, 21].

The CONSORT reporting guideline was developed in order to improve the reporting of RCTs, making it easier to ascertain what was done, identify possible sources of bias, and evaluate the reliability of a study [22, 23]. In general, the adoption of the CONSORT checklist has improved the reporting of RCTs in the medical literature, but there are still reporting deficits [24, 25]. In veterinary medicine the REFLECT statement is also available, which is an extension to the CONSORT reporting guideline specifically developed for RCTs involving livestock [26, 27]. Strict adherence to such reporting guidelines [28] should have reduced all the ‘unclear’ assessments of bias made in this study and would have allowed us to identify the funding source of all the trials. Most importantly, this would allow more reliable assessments of treatment efficacies to be made, meaning more effective translation of evidence into clinical practice. A recent survey assessing the awareness of reporting guidelines amongst veterinary editors reported that 35.1% of journal editors said reporting guidelines were referred to in their instructions to authors [29]. An improvement in the endorsement of reporting guidelines by journals could help to improve the reporting quality of the veterinary clinical trial literature as it has done for medicine.

A significant limitation of this study is that there were a relatively small number of trials included in the analysis, and due to the large proportion of pharmaceutical trials in the sample (68%), the groups for comparison were unbalanced and the non-pharmaceutical group small. Another, larger study would be extremely beneficial in assessing the presence of sponsorship bias in the veterinary clinical trials literature. In particular, an exploration of potential differences between species, or between companion animal versus production animals, warrants further investigation with larger sample sizes (no significant differences were found in the current study, see Table 2). Results of this type of study can be very dependent on the methods, including what types of studies are included (e.g. we have only included pharmaceutical interventions), which outcome classifications are used, the way in which outcomes are extracted (e.g. we did not include results for outcomes which were not mentioned in the materials and methods) and how funding categories are divided, meaning results across studies could be very different. Another limitation of this study is that the authors were not blinded to any manuscript details during data extraction potentially leading to biased interpretation. The lack of inclusion of efficacy studies where multiple doses of the test treatment were used is another significant limitation of the study. On balance it was felt that inclusion of these could potentially skew the results due to multiple entries for the trial by including each dose, or selecting only one of the doses. The inclusion of multiple trials within one publication may also skew results, as the methods, and therefore assessment of quality, tend to be identical for all the included trials. As most multiple trial papers were in the pharmaceutical category, this could potentially lead to clustering of information. Of particular influence in this study were RCTs assessing anthelmintic agents as these often contained multiple similar trials with an overwhelming proportion of positive outcomes. As they fulfilled our initial inclusion criteria they remained in our sample but their impact on the overall results may be substantial. The subjective assignment of a single outcome result for an outcome which was assessed at multiple time points is another limitation which was necessary for practicality. Limits to the initial sample size were needed due to cost and time constraints; a single calendar year search in PubMed was chosen to give a representative, recent sample of trials, rather than selecting certain journals to search. Using PubMed also allowed us to search by publication type. Not including studies unavailable in English was a necessary cost and time limitation but only one paper was excluded on this basis so this is unlikely to have affected the study outcomes.

## Conclusions

This study found a positive association between pharmaceutical funding or involvement and increased positive outcome reporting. Consistently poor reporting of trials, including non-identification of funding source was identified, which hinders the assessment and use of the limited evidence available to the profession.

## Additional files

Additional file 1: Cochrane (http://www.cochrane.org/glossary/) definitions of types of bias. Written descriptions of the definitions of the Cochrane types of bias. (DOCX 14 kb)

Additional file 2: Table S1. Reasons for exclusions of RCTs involving pharmaceutical agents from analysis. Table containing numbers of trials excluded for each reason organised in species groups. (DOCX 14 kb)

Additional file 3: References for all papers included in the analysis within this study (single dose efficacy studies of pharmaceutical interventions in cats, dogs, horses, cattle or sheep published in 2011). List of references in word. (DOCX 26 kb)

## Abbreviations

CONSORT: Consolidated Standards for Reporting Trials
NF: Group of trials for which no funding source was stated
NP: Group of trials for which non-pharmaceutical funding was stated
P: Group of trials for which pharmaceutical funding/involvement was stated
RCT: Randomised controlled trial
