# Supplementary material for: Sponsorship bias and quality of randomised controlled trials in veterinary medicine
Source: BMC Vet Res. 2017 Aug 14;13:234. doi: 10.1186/s12917-017-1146-9 (PMC5557072; doi:10.1186/s12917-017-1146-9)
Supplement: Supplementary file 1 — Cochrane (http://www.cochrane.org/glossary/) definitions of types of bias. Written descriptions of the definitions of the Cochrane types of bias. (DOCX 14 kb) [file 12917_2017_1146_MOESM1_ESM.docx]

**Additional file 1.**

**Cochrane (**<http://www.cochrane.org/glossary/>) **definitions of types of bias**

Selection bias: Systematic differences between comparison groups in prognosis or responsiveness to treatment. Random allocation with adequate concealment of allocation protects against selection bias. Other means of selecting who receives the intervention are more prone to bias because decisions may be related to prognosis or responsiveness to treatment.

Performance bias: Systematic differences between intervention groups in care provided apart from the intervention being evaluated. For example, if participants know they are in the control group, they may be more likely to use other forms of care. If care providers are aware of the group a particular participant is in, they might act differently. Blinding of study participants (both the recipients and providers of care) is used to protect against performance bias.

Detection bias: Systematic difference between comparison groups in how outcomes are ascertained, diagnosed or verified.

Attrition bias: Systematic differences between comparison groups in withdrawals or exclusions of participants from the results of a study. For example, participants may drop out of a study because of side effects of an intervention, and excluding these participants from the analysis could result in an overestimate of the effectiveness of the intervention, especially when the proportion dropping out varies by treatment group.

Reporting bias: A bias caused by only a subset of all the relevant data being available. The publication of research can depend on the nature and direction of the study results. Studies in which an intervention is not found to be effective are sometimes not published. Because of this, systematic reviews that fail to include unpublished studies may overestimate the true effect of an intervention. In addition, a published report might present a biased set of results (e.g. only outcomes or sub-groups where a statistically significant difference was found.
