# Supplementary material for: Sponsorship bias and quality of randomised controlled trials in veterinary medicine
Source: BMC Vet Res. 2017 Aug 14;13:234. doi: 10.1186/s12917-017-1146-9 (PMC5557072; doi:10.1186/s12917-017-1146-9)
Supplement: Supplementary file 2 — Reasons for exclusions of RCTs involving pharmaceutical agents from analysis. Table containing numbers of trials excluded for each reason organised in species groups. (DOCX 14 kb) [file 12917_2017_1146_MOESM2_ESM.docx]

**Additional file 2: Table S1.**

**Reasons for exclusions of RCTs involving pharmaceutical agents from analysis**

|  | **Number of cat papers (trials)** | **Number of dog papers (trials)** | **Number of horse papers (trials)** | **Number of cattle papers (trials)** | **Number of sheep papers (trials)** | **Number of total papers (trials)** |
| --- | --- | --- | --- | --- | --- | --- |
| Papers including pharmaceutical agent RCTs | 17 | 49 | 28 | 61 | 17 | 172 |
| Papers analysed | 8 (9 trials) | 28 (44 trials) | 11 (11 trials) | 32 (36 trials) | 7 (26 trials) | 86 (126 trials) |
| **Reasons for exclusion of papers** |  |  |  |  |  |  |
| Treatment of interest could not be identified | 2 | 5 | 5 | 12 | 4 | 28 |
| Pharmacokinetic/pharmacodynamic studies | 1 | 2 | 4 | 2 | 1 | 10 |
| Dose finding studies | 3 | 1 | 1 | 1 | 0 | 6 |
| Multiple doses of the treatment of interest were used | 1 | 8 | 2 | 2 | 3 | 16 |
| Safety studies in healthy animals | 1 | 3 | 1 | 1 | 0 | 6 |
| Physiological effect studies | 1 | 0 | 1 | 8 | 2 | 12 |
| Resistance to pharmaceutical studies | 0 | 0 | 0 | 1 | 0 | 1 |
| Route of administration | 0 | 2 | 1 | 1 | 0 | 4 |
| Timing of administration | 0 | 0 | 1 | 1 | 0 | 2 |
| Not available in English | 0 | 0 | 1 | 0 | 0 | 1 |
