# Supplementary material for: Sponsorship bias and quality of randomised controlled trials in veterinary medicine
Source: BMC Vet Res. 2017 Aug 14;13:234. doi: 10.1186/s12917-017-1146-9 (PMC5557072; doi:10.1186/s12917-017-1146-9)
Supplement: Supplementary file 3 — References for all papers included in the analysis within this study (single dose efficacy studies of pharmaceutical interventions in cats, dogs, horses, cattle or sheep published in 2011). List of references in word. (DOCX 26 kb) [file 12917_2017_1146_MOESM3_ESM.docx]

**Additional file 3.**

**References for all papers included in the analysis within this study (single dose efficacy studies of pharmaceutical interventions in cats, dogs, horses, cattle or sheep published in 2011)**

1. Abelson AL, Armitage-Chan E, Lindsey JC, Wetmore LA: A comparison of epidural morphine with low dose bupivacaine versus epidural morphine alone on motor and respiratory function in dogs following splenectomy. Veterinary anaesthesia and analgesia 2011, 38(3):213-223.

2. Agaoglu AR, Schafer-Somi S, Kaya D, Kucukaslan I, Emre B, Gultiken N, Mulazimoglu BS, Colak A, Aslan S: The intravaginal application of misoprostol improves induction of abortion with aglepristone. Theriogenology 2011, 76(1):74-82.

3. Aguado D, Benito J, Gomez de Segura IA: Reduction of the minimum alveolar concentration of isoflurane in dogs using a constant rate of infusion of lidocaine-ketamine in combination with either morphine or fentanyl. Veterinary journal (London, England : 1997) 2011, 189(1):63-66.

4. Allen KJ, Rogan D, Finlay BB, Potter AA, Asper DJ: Vaccination with type III secreted proteins leads to decreased shedding in calves after experimental infection with Escherichia coli O157. Canadian journal of veterinary research = Revue canadienne de recherche veterinaire 2011, 75(2):98-105.

5. Altreuther G, Gasda N, Adler K, Hellmann K, Thurieau H, Schimmel A, Hutchens D, Krieger KJ: Field evaluations of the efficacy and safety of Emodepside plus toltrazuril (Procox(R) oral suspension for dogs) against naturally acquired nematode and Isospora spp. infections in dogs. Parasitology research 2011, 109 Suppl 1:S21-28.

6. Altreuther G, Gasda N, Schroeder I, Joachim A, Settje T, Schimmel A, Hutchens D, Krieger KJ: Efficacy of emodepside plus toltrazuril suspension (Procox((R)) oral suspension for dogs) against prepatent and patent infection with Isospora canis and Isospora ohioensis-complex in dogs. Parasitology research 2011, 109 Suppl 1:S9-20.

7. Avendano-Reyes L, Macias-Cruz U, Alvarez-Valenzuela FD, Aguila-Tepato E, Torrentera-Olivera NG, Soto-Navarro SA: Effects of zilpaterol hydrochloride on growth performance, carcass characteristics, and wholesale cut yield of hair-breed ewe lambs consuming feedlot diets under moderate environmental conditions. Journal of animal science 2011, 89(12):4188-4194.

8. Baggott D, Casartelli A, Fraisse F, Manavella C, Marteau R, Rehbein S, Wiedemann M, Yoon S: Demonstration of the metaphylactic use of gamithromycin against bacterial pathogens associated with bovine respiratory disease in a multicentre farm trial. The Veterinary record 2011, 168(9):241.

9. Bergamasco L, Coetzee JF, Gehring R, Murray L, Song T, Mosher RA: Effect of intravenous sodium salicylate administration prior to castration on plasma cortisol and electroencephalography parameters in calves. Journal of veterinary pharmacology and therapeutics 2011, 34(6):565-576.

10. Bettschart-Wolfensberger R, Dicht S, Vullo C, Frotzler A, Kuemmerle JM, Ringer SK: A clinical study on the effect in horses during medetomidine-isoflurane anaesthesia, of butorphanol constant rate infusion on isoflurane requirements, on cardiopulmonary function and on recovery characteristics. Veterinary anaesthesia and analgesia 2011, 38(3):186-194.

11. Beugnet F, Doyle V, Murray M, Chalvet-Monfray K: Comparative efficacy on dogs of a single topical treatment with the pioneer fipronil/(S)-methoprene and an oral treatment with spinosad against Ctenocephalides felis. Parasite (Paris, France) 2011, 18(4):325-331.

12. Bryan MA, Heuer C, Emslie FR: The comparative efficacy of two long-acting dry-cow cephalonium products in curing and preventing intramammary infections. New Zealand veterinary journal 2011, 59(4):166-173.

13. Buckley GJ, Rozanski EA, Rush JE: Randomized, blinded comparison of epinephrine and vasopressin for treatment of naturally occurring cardiopulmonary arrest in dogs. Journal of veterinary internal medicine / American College of Veterinary Internal Medicine 2011, 25(6):1334-1340.

14. Cadot P, Hensel P, Bensignor E, Hadjaje C, Marignac G, Beco L, Fontaine J, Jamet JF, Georgescu G, Campbell K et al: Masitinib decreases signs of canine atopic dermatitis: a multicentre, randomized, double-blind, placebo-controlled phase 3 trial. Veterinary dermatology 2011, 22(6):554-564.

15. Camargo JB, Steagall PV, Minto BW, Lorena SE, Mori ES, Luna SP: Post-operative analgesic effects of butorphanol or firocoxib administered to dogs undergoing elective ovariohysterectomy. Veterinary anaesthesia and analgesia 2011, 38(3):252-259.

16. Cohn LA, Birkenheuer AJ, Brunker JD, Ratcliff ER, Craig AW: Efficacy of atovaquone and azithromycin or imidocarb dipropionate in cats with acute cytauxzoonosis. Journal of veterinary internal medicine / American College of Veterinary Internal Medicine 2011, 25(1):55-60.

17. Congdon JM, Marquez M, Niyom S, Boscan P: Evaluation of the sedative and cardiovascular effects of intramuscular administration of dexmedetomidine with and without concurrent atropine administration in dogs. Journal of the American Veterinary Medical Association 2011, 239(1):81-89.

18. Davey RB, Pound JM, Klavons JA, Lohmeyer KH, Freeman JM, Perez de Leon AA, Miller RJ: Efficacy and blood sera analysis of a long-acting formulation of moxidectin against Rhipicephalus (Boophilus) microplus (Acari: Ixodidae) on treated cattle. Journal of medical entomology 2011, 48(2):314-321.

19. Dodd CC, Renter DG, Thomson DU, Nagaraja TG: Evaluation of the effects of a commercially available Salmonella Newport siderophore receptor and porin protein vaccine on fecal shedding of Salmonella bacteria and health and performance of feedlot cattle. American journal of veterinary research 2011, 72(2):239-247.

20. Faya M, Carranza A, Priotto M, Graiff D, Zurbriggen G, Diaz JD, Gobello C: Long-term melatonin treatment prolongs interestrus, but does not delay puberty, in domestic cats. Theriogenology 2011, 75(9):1750-1754.

21. Felix TL, Loerch SC: Effects of haylage and monensin supplementation on performance, carcass characteristics, and ruminal metabolism of feedlot cattle fed diets containing 60% dried distillers grains. Journal of animal science 2011, 89(8):2614-2623.

22. Fischer Y, Ritz S, Weber K, Sauter-Louis C, Hartmann K: Randomized, placebo controlled study of the effect of propentofylline on survival time and quality of life of cats with feline infectious peritonitis. Journal of veterinary internal medicine / American College of Veterinary Internal Medicine 2011, 25(6):1270-1276.

23. Fourie JJ, Beugnet F, Ollagnier C, Pollmeier MG: Study of the sustained speed of kill of the combination of fipronil/amitraz/(S)-methoprene and the combination of imidacloprid/permethrin against Dermacentor reticulatus, the European dog tick. Parasite (Paris, France) 2011, 18(4):319-323.

24. Friedman E, Voet H, Reznikov D, Dagoni I, Roth Z: Induction of successive follicular waves by gonadotropin-releasing hormone and prostaglandin F(2alpha) to improve fertility of high-producing cows during the summer and autumn. Journal of dairy science 2011, 94(5):2393-2402.

25. Gabriel HG, Wallenhorst S, Dietrich E, Holtz W: The effect of prostaglandin F(2alpha) administration at the time of insemination on the pregnancy rate of dairy cows. Animal reproduction science 2011, 123(1-2):1-4.

26. Geary TW, Wells KJ, deAvila DM, deAvila J, Conforti VA, McLean DJ, Roberts AJ, Waterman RW, Reeves JJ: Effects of immunization against luteinizing hormone-releasing hormone and treatment with trenbolone acetate on reproductive function of beef bulls and steers. Journal of animal science 2011, 89(7):2086-2095.

27. Gordon-Evans WJ, Dunning D, Johnson AL, Knap KE: Effect of the use of carprofen in dogs undergoing intense rehabilitation after lateral fabellar suture stabilization. Journal of the American Veterinary Medical Association 2011, 239(1):75-80.

28. Gruet P, Seewald W, King JN: Evaluation of subcutaneous and oral administration of robenacoxib and meloxicam for the treatment of acute pain and inflammation associated with orthopedic surgery in dogs. American journal of veterinary research 2011, 72(2):184-193.

29. Habing GG, Neuder LM, Raphael W, Piper-Youngs H, Kaneene JB: Efficacy of oral administration of a modified-live Salmonella Dublin vaccine in calves. Journal of the American Veterinary Medical Association 2011, 238(9):1184-1190.

30. Hardie EM, Lascelles BD, Meuten T, Davidson GS, Papich MG, Hansen BD: Evaluation of intermittent infusion of bupivacaine into surgical wounds of dogs postoperatively. Veterinary journal (London, England : 1997) 2011, 190(2):287-289.

31. Hellmann K, Heine J, Braun G, Paran-Dobesova R, Svobodova V: Evaluation of the therapeutic and preventive efficacy of 2.5 % moxidectin / 10 % imidacloprid (Advocate((R)), Bayer animal health) in dogs naturally infected or at risk of natural infection by Dirofilaria repens. Parasitology research 2011, 109 Suppl 1:S77-86.

32. Hennet PR, Camy GA, McGahie DM, Albouy MV: Comparative efficacy of a recombinant feline interferon omega in refractory cases of calicivirus-positive cats with caudal stomatitis: a randomised, multi-centre, controlled, double-blind study in 39 cats. Journal of feline medicine and surgery 2011, 13(8):577-587.

33. Hermo GA, Turic E, Angelico D, Scursoni AM, Gomez DE, Gobello C, Alonso DF: Effect of adjuvant perioperative desmopressin in locally advanced canine mammary carcinoma and its relation to histologic grade. Journal of the American Animal Hospital Association 2011, 47(1):21-27.

34. Heuwieser W, Iwersen M, Goetze L: Efficacy of carprofen on conception rates in lactating dairy cows after subcutaneous or intrauterine administration at the time of breeding. Journal of dairy science 2011, 94(1):146-151.

35. Horohov DW, Loynachan AT, Page AE, Hughes K, Timoney JF, Fettinger M, Hatch T, Spaulding JG, McMichael J: The use of streptolysin O (SLO) as an adjunct therapy for Rhodococcus equi pneumonia in foals. Veterinary microbiology 2011, 154(1-2):156-162.

36. Johnston TP, Mondal P, Pal D, MacGee S, Stromberg AJ, Alur H: Canine periodontal disease control using a clindamycin hydrochloride gel. Journal of veterinary dentistry 2011, 28(4):224-229.

37. Jonsson NN, Piper EK, Gray CP, Deniz A, Constantinoiu CC: Efficacy of toltrazuril 5 % suspension against Eimeria bovis and Eimeria zuernii in calves and observations on the associated immunopathology. Parasitology research 2011, 109 Suppl 1:S113-128.

38. Kasravi R, Bolourchi M, Farzaneh N, Seifi HA, Barin A, Hovareshti P, Gharagozlou F: Efficacy of conventional and extended intra-mammary treatment of persistent sub-clinical mastitis with cefquinome in lactating dairy cows. Tropical animal health and production 2011, 43(6):1203-1210.

39. Kilpinen S, Spillmann T, Syrja P, Skrzypczak T, Louhelainen M, Westermarck E: Effect of tylosin on dogs with suspected tylosin-responsive diarrhea: a placebo-controlled, randomized, double-blinded, prospective clinical trial. Acta veterinaria Scandinavica 2011, 53:26.

40. Kloppel H, Leece EA: Comparison of ketamine and alfaxalone for induction and maintenance of anaesthesia in ponies undergoing castration. Veterinary anaesthesia and analgesia 2011, 38(1):37-43.

41. Knights M, Ramgattie R, Siew N, Singh-Knights D, Bourne G: Effectiveness of a short-term treatment with progesterone injections on synchrony of lambing and fertility in tropical hair sheep. Animal reproduction science 2011, 126(1-2):70-75.

42. Lawrence TE, Gasch CA, Hutcheson JP, Hodgen JM: Zilpaterol improves feeding performance and fabrication yield of concentrate-finished cull cows. Journal of animal science 2011, 89(7):2170-2175.

43. Levy JK, Friary JA, Miller LA, Tucker SJ, Fagerstone KA: Long-term fertility control in female cats with GonaCon, a GnRH immunocontraceptive. Theriogenology 2011, 76(8):1517-1525.

44. Little PR, Hodge A, Maeder SJ, Wirtherle NC, Nicholas DR, Cox GG, Conder GA: Efficacy of a combined oral formulation of derquantel-abamectin against the adult and larval stages of nematodes in sheep, including anthelmintic-resistant strains. Veterinary parasitology 2011, 181(2-4):180-193.

45. Ma J, Shi N, Jiang CG, Lin YZ, Wang XF, Wang S, Lv XL, Zhao LP, Shao YM, Kong XG et al: A proviral derivative from a reference attenuated EIAV vaccine strain failed to elicit protective immunity. Virology 2011, 410(1):96-106.

46. Macrina AL, Tozer PR, Kensinger RS: Induced lactation in pubertal heifers: efficacy, response to bovine somatotropin, and profitability. Journal of dairy science 2011, 94(3):1355-1364.

47. Marino CT, Otero WG, Rodrigues PH, Dicostanzo A, Millen DD, Pacheco RL, Dilorenzo N, Martins CL, Arrigoni MD: Effects of adding polyclonal antibody preparations on ruminal fermentation patterns and digestibility of cows fed different energy sources. Journal of animal science 2011, 89(10):3228-3235.

48. Marquezini GH, Dahlen CR, Bird SL, Lamb GC: Administration of human chorionic gonadotropin to suckled beef cows before ovulation synchronization and fixed-time insemination: replacement of gonadotropin-releasing hormone with human chorionic gonadotropin. Journal of animal science 2011, 89(10):3030-3039.

49. Martins JP, Policelli RK, Neuder LM, Raphael W, Pursley JR: Effects of cloprostenol sodium at final prostaglandin F2alpha of Ovsynch on complete luteolysis and pregnancy per artificial insemination in lactating dairy cows. Journal of dairy science 2011, 94(6):2815-2824.

50. McArt JA, Nydam DV, Ospina PA, Oetzel GR: A field trial on the effect of propylene glycol on milk yield and resolution of ketosis in fresh cows diagnosed with subclinical ketosis. Journal of dairy science 2011, 94(12):6011-6020.

51. McClure S, Sibert G, Hallberg J, Bade D: Efficacy of a 2-dose regimen of a sustained release ceftiofur suspension in horses with Streptococcus equi subsp. zooepidemicus bronchopneumonia. Journal of veterinary pharmacology and therapeutics 2011, 34(5):442-447.

52. Mellett AM, Nakamura RK, Bianco D: A prospective study of clopidogrel therapy in dogs with primary immune-mediated hemolytic anemia. Journal of veterinary internal medicine / American College of Veterinary Internal Medicine 2011, 25(1):71-75.

53. Merino O, Almazan C, Canales M, Villar M, Moreno-Cid JA, Estrada-Pena A, Kocan KM, de la Fuente J: Control of Rhipicephalus (Boophilus) microplus infestations by the combination of subolesin vaccination and tick autocidal control after subolesin gene knockdown in ticks fed on cattle. Vaccine 2011, 29(12):2248-2254.

54. Meyers-Brown G, Bidstrup LA, Famula TR, Colgin M, Roser JF: Treatment with recombinant equine follicle stimulating hormone (reFSH) followed by recombinant equine luteinizing hormone (reLH) increases embryo recovery in superovulated mares. Animal reproduction science 2011, 128(1-4):52-59.

55. Morton CM, Grant D, Johnston L, Letellier IM, Narbe R: Clinical evaluation of meloxicam versus ketoprofen in cats suffering from painful acute locomotor disorders. Journal of feline medicine and surgery 2011, 13(4):237-243.

56. O'Connor AM, Brace S, Gould S, Dewell R, Engelken T: A randomized clinical trial evaluating a farm-of-origin autogenous Moraxella bovis vaccine to control infectious bovine keratoconjunctivis (pinkeye) in beef cattle. Journal of veterinary internal medicine / American College of Veterinary Internal Medicine 2011, 25(6):1447-1453.

57. Olivera-Muzante J, Fierro S, Lopez V, Gil J: Comparison of prostaglandin- and progesterone-based protocols for timed artificial insemination in sheep. Theriogenology 2011, 75(7):1232-1238.

58. Olsen L, Bondesson U, Brostrom H, Olsson U, Mazogi B, Sundqvist M, Tjalve H, Ingvast-Larsson C: Pharmacokinetics and effects of cetirizine in horses with insect bite hypersensitivity. Veterinary journal (London, England : 1997) 2011, 187(3):347-351.

59. Pang WY, Earley B, Murray M, Sweeney T, Gath V, Crowe MA: Banding or Burdizzo castration and carprofen administration on peripheral leukocyte inflammatory cytokine transcripts. Research in veterinary science 2011, 90(1):127-132.

60. Parr SL, Chung KY, Hutcheson JP, Nichols WT, Yates DA, Streeter MN, Swingle RS, Galyean ML, Johnson BJ: Dose and release pattern of anabolic implants affects growth of finishing beef steers across days on feed. Journal of animal science 2011, 89(3):863-873.

61. Pasa S, Voyvoda H, Karagenc T, Atasoy A, Gazyagci S: Failure of combination therapy with imidocarb dipropionate and toltrazuril to clear Hepatozoon canis infection in dogs. Parasitology research 2011, 109(3):919-926.

62. Pinard CL, Gauvin D, Moreau M, Martel-Pelletier J, Pelletier JP, Troncy E: Measurements of canine aqueous humor inflammatory mediators and the effect of carprofen following anterior chamber paracentesis. Veterinary ophthalmology 2011, 14(5):296-303.

63. Psatha E, Alibhai HI, Jimenez-Lozano A, Armitage-Chan E, Brodbelt DC: Clinical efficacy and cardiorespiratory effects of alfaxalone, or diazepam/fentanyl for induction of anaesthesia in dogs that are a poor anaesthetic risk. Veterinary anaesthesia and analgesia 2011, 38(1):24-36.

64. Redmond JS, Macedo GG, Velez IC, Caraty A, Williams GL, Amstalden M: Kisspeptin activates the hypothalamic-adenohypophyseal-gonadal axis in prepubertal ewe lambs. Reproduction (Cambridge, England) 2011, 141(4):541-548.

65. Reist M, Forbes AB, Bonfanti M, Beretta W, Pfister K: Effect of eprinomectin treatment on milk yield and quality in dairy cows in South Tyrol, Italy. The Veterinary record 2011, 168(18):484.

66. Santos LC, Ludders JW, Erb HN, Martin-Flores M, Basher KL, Kirch P: A randomized, blinded, controlled trial of the antiemetic effect of ondansetron on dexmedetomidine-induced emesis in cats. Veterinary anaesthesia and analgesia 2011, 38(4):320-327.

67. Sawalha MN, Kridli RT, Jawasreh KI, Meza-Herrera CA: The use of melatonin and progestagen-eCG to initiate reproductive activity in prepuberal Awassi ewe lambs. Tropical animal health and production 2011, 43(7):1345-1350.

68. Schauer SN, Briant C, Ottogalli M, Decourt C, Handel IG, Donadeu FX: Supplementation of equine early spring transitional follicles with luteinizing hormone stimulates follicle growth but does not restore steroidogenic activity. Theriogenology 2011, 75(6):1076-1084.

69. Schauvliege S, Marcilla MG, Verryken K, Duchateau L, Devisscher L, Gasthuys F: Effects of a constant rate infusion of detomidine on cardiovascular function, isoflurane requirements and recovery quality in horses. Veterinary anaesthesia and analgesia 2011, 38(6):544-554.

70. Schimmel A, Schroeder I, Altreuther G, Settje T, Charles S, Wolken S, Kok DJ, Ketzis J, Young D, Hutchens D et al: Efficacy of emodepside plus toltrazuril (Procox((R)) oral suspension for dogs) against Toxocara canis, Uncinaria stenocephala and Ancylostoma caninum in dogs. Parasitology research 2011, 109 Suppl 1:S1-8.

71. Schukken YH, Bennett GJ, Zurakowski MJ, Sharkey HL, Rauch BJ, Thomas MJ, Ceglowski B, Saltman RL, Belomestnykh N, Zadoks RN: Randomized clinical trial to evaluate the efficacy of a 5-day ceftiofur hydrochloride intramammary treatment on nonsevere gram-negative clinical mastitis. Journal of dairy science 2011, 94(12):6203-6215.

72. Schuller S, Van Israel N, Vanbelle S, Clercx C, McEntee K: Lack of efficacy of low-dose spironolactone as adjunct treatment to conventional congestive heart failure treatment in dogs. Journal of veterinary pharmacology and therapeutics 2011, 34(4):322-331.

73. Siedek EM, Schmidt H, Sture GH, Raue R: Vaccination with canine parvovirus type 2 (CPV-2) protects against challenge with virulent CPV-2b and CPV-2c. Berliner und Munchener tierarztliche Wochenschrift 2011, 124(1-2):58-64.

74. Snyder DE, Wiseman S, Bowman DD, McCall JW, Reinemeyer CR: Assessment of the effectiveness of a combination product of spinosad and milbemycin oxime on the prophylaxis of canine heartworm infection. Veterinary parasitology 2011, 180(3-4):262-266.

75. Sturgill TL, Giguere S, Franklin RP, Cohen ND, Hagen J, Kalyuzhny AE: Effects of inactivated parapoxvirus ovis on the cumulative incidence of pneumonia and cytokine secretion in foals on a farm with endemic infections caused by Rhodococcus equi. Veterinary immunology and immunopathology 2011, 140(3-4):237-243.

76. Teske E, Rutteman GR, Kirpenstein J, Hirschberger J: A randomized controlled study into the efficacy and toxicity of pegylated liposome encapsulated doxorubicin as an adjuvant therapy in dogs with splenic haemangiosarcoma. Veterinary and comparative oncology 2011, 9(4):283-289.

77. Thiry J, Rubion S, Sarasola P, Bonnier M, Hartmann M, de Haas V: Efficacy and safety of a new 450 mg/ml florfenicol formulation administered intramuscularly in the treatment of bacterial bovine respiratory disease. The Veterinary record 2011, 169(20):526.

78. Thomasy SM, Lim CC, Reilly CM, Kass PH, Lappin MR, Maggs DJ: Evaluation of orally administered famciclovir in cats experimentally infected with feline herpesvirus type-1. American journal of veterinary research 2011, 72(1):85-95.

79. Trotz-Williams LA, Jarvie BD, Peregrine AS, Duffield TF, Leslie KE: Efficacy of halofuginone lactate in the prevention of cryptosporidiosis in dairy calves. The Veterinary record 2011, 168(19):509.

80. Unterer S, Strohmeyer K, Kruse BD, Sauter-Louis C, Hartmann K: Treatment of aseptic dogs with hemorrhagic gastroenteritis with amoxicillin/clavulanic acid: a prospective blinded study. Journal of veterinary internal medicine / American College of Veterinary Internal Medicine 2011, 25(5):973-979.

81. VanLeeuwen JA, Greenwood S, Clark F, Acorn A, Markham F, McCarron J, O'Handley R: Monensin use against Neospora caninum challenge in dairy cattle. Veterinary parasitology 2011, 175(3-4):372-376.

82. Vasconcelos JL, Sa Filho OG, Justolin PL, Morelli P, Aragon FL, Veras MB, Soriano S: Effects of postbreeding gonadotropin treatments on conception rates of lactating dairy cows subjected to timed artificial insemination or embryo transfer in a tropical environment. Journal of dairy science 2011, 94(1):223-234.

83. Veronesi F, Diaferia M, Viola O, Fioretti DP: Long-term effect of toltrazuril on growth performances of dairy heifers and beef calves exposed to natural Eimeria zuernii and Eimeria bovis infections. Veterinary journal (London, England : 1997) 2011, 190(2):296-299.

84. Villalba M, Santiago I, Gomez de Segura IA: Effects of constant rate infusion of lidocaine and ketamine, with or without morphine, on isoflurane MAC in horses. Equine veterinary journal 2011, 43(6):721-726.

85. von Krueger X, Heuwieser W: Effect of CIDR(R) on 4-day-service-rate, pregnancy rate and vaginal irritation in dairy heifers. Tierarztliche Praxis Ausgabe G, Grosstiere/Nutztiere 2011, 39(5):277-280.

86. Wall R, Bates P: Sheep scab control using trans-cinnamic acid. Veterinary parasitology 2011, 175(1-2):129-134.
